# Bronchocentric Granulomatosis Mimicking Diffuse Malignancy: Reticulonodular Lung Disease With Widespread 2-Deoxy-2-[¹⁸F]fluoro-D-Glucose Uptake

**DOI:** 10.7759/cureus.101900

**Published:** 2026-01-20

**Authors:** Akane Ozawa, Kota Yokoyama, Haruhiko Furusawa, Takashi Ito, Ukihide Tateishi

**Affiliations:** 1 Diagnostic Radiology and Nuclear Medicine, Institute of Science Tokyo, Tokyo, JPN; 2 Diagnostic Radiology and Nuclear Medicine, Tokyo Medical and Dental University, Tokyo, JPN; 3 Respiratory Medicine, Institute of Science Tokyo, Tokyo, JPN; 4 Pathology, Institute of Science Tokyo, Tokyo, JPN

**Keywords:** bronchocentric granulomatosis, diffuse reticulonodular opacities, fdg pet/ct, fungal infection, immunologic response

## Abstract

Bronchocentric granulomatosis (BCG) is a rare airway-centered granulomatous disorder and is regarded as an immunologically mediated reaction rather than an active infectious process. Its imaging findings are heterogeneous and often non-specific, frequently mimicking malignant or infectious lung diseases.

We report a 63-year-old woman who presented with persistent fever and diffuse bilateral reticulonodular opacities on chest computed tomography (CT), an uncommon imaging manifestation of BCG. 2-Deoxy-2-[¹⁸F]fluoro-D-glucose positron emission tomography/CT ([¹⁸F]FDG-PET/CT) demonstrated diffuse pulmonary uptake without extrapulmonary involvement, raising concern for malignant disease. Repeated bronchoscopic biopsies were non-diagnostic, and a surgical lung biopsy revealed bronchocentric granulomatous inflammation with only minimal fungal elements, leading to a diagnosis of BCG.

Diffuse reticulonodular CT patterns and [¹⁸F]FDG-PET findings in BCG have been rarely reported. This case highlights that BCG can present with metabolically active diffuse lung disease and be mistaken for malignancy. Awareness of this rare entity and its atypical imaging features is important for radiologists when interpreting diffuse nodular lung disease with inconclusive microbiological results.

## Introduction

Bronchocentric granulomatosis (BCG) is a rare pulmonary granulomatous disorder characterized histopathologically by necrotizing granulomas centered on bronchi and bronchioles. It is widely regarded as an immunologically mediated reaction to fungal or other microbial antigens rather than an active infectious process, and the causative organisms are often absent or detected only in minimal quantities on histopathological examination [1-3].

Radiologically, BCG demonstrates heterogeneous manifestations, most commonly reported as mass-like lesions, nodules, or focal consolidation [4]. Diffuse reticulonodular opacities are rarely described and represent an uncommon imaging presentation of this disease [4-10]. Furthermore, the metabolic characteristics of BCG on 2-deoxy-2-[¹⁸F]fluoro-D-glucose positron emission tomography/computed tomography ([¹⁸F]FDG-PET/CT) remain poorly defined, with only a single case previously reported in the literature [5].

Here, we report a rare case of BCG presenting with diffuse bilateral reticulonodular opacities and diffuse pulmonary [¹⁸F]FDG uptake, despite only minimal fungal elements identified on histopathology. This case provides important radiologic-pathologic correlation and offers insight into the immunopathological nature of BCG, emphasizing the limitations of imaging and microbiological studies alone in establishing a definitive diagnosis.

## Case presentation

A 63-year-old woman presented with a persistent fever lasting several weeks that was unresponsive to antibiotic therapy. She had no remarkable history of asthma or allergic bronchopulmonary aspergillosis. Physical examination was unremarkable, and routine laboratory investigations showed no specific abnormalities suggestive of infection or autoimmune disease.

**Figure 1:**
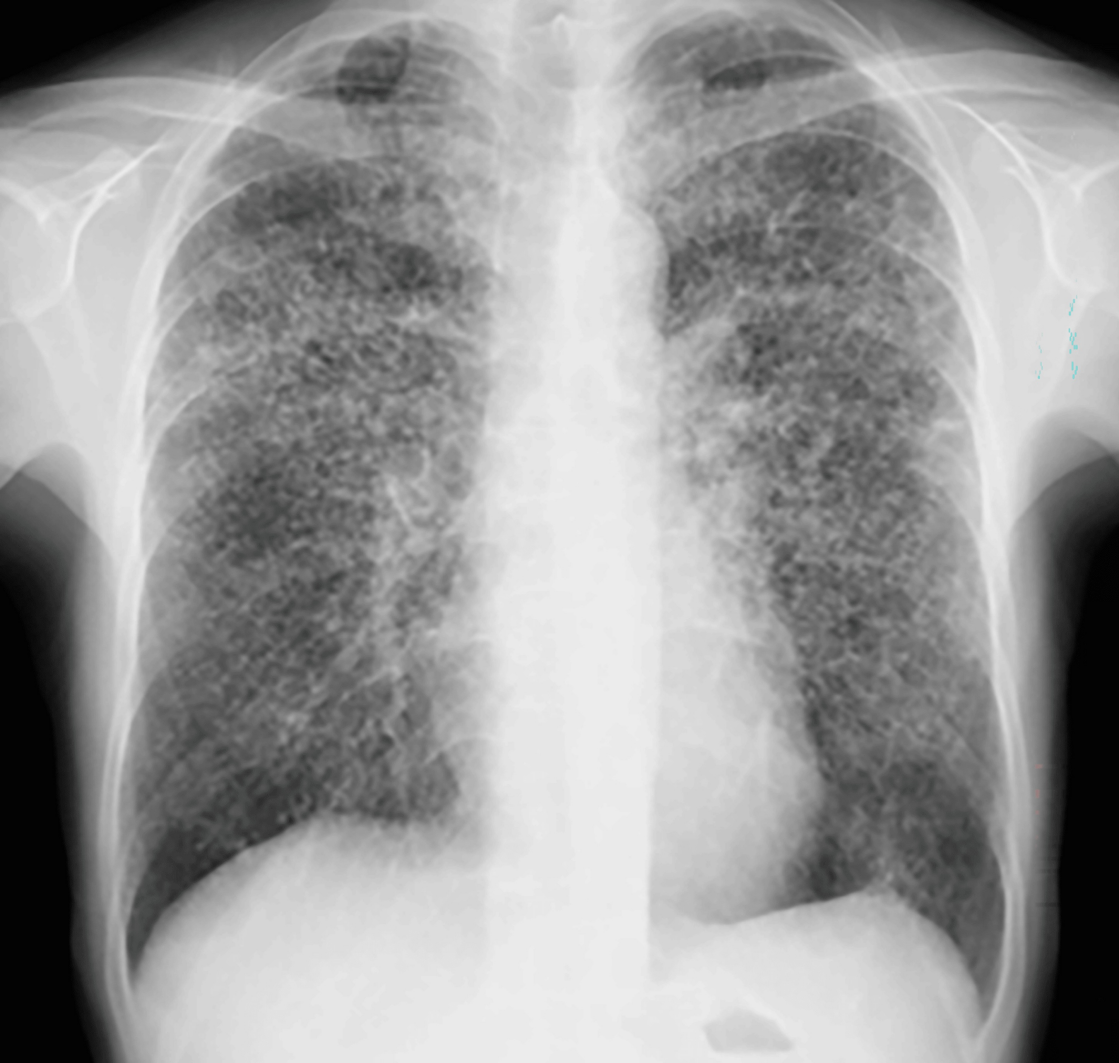
Chest radiograph (upright posteroanterior view) The chest radiograph demonstrates diffuse bilateral reticulonodular opacities throughout both lung fields.

Chest radiography demonstrated diffuse bilateral reticulonodular opacities (Figure 1).

**Figure 2:**
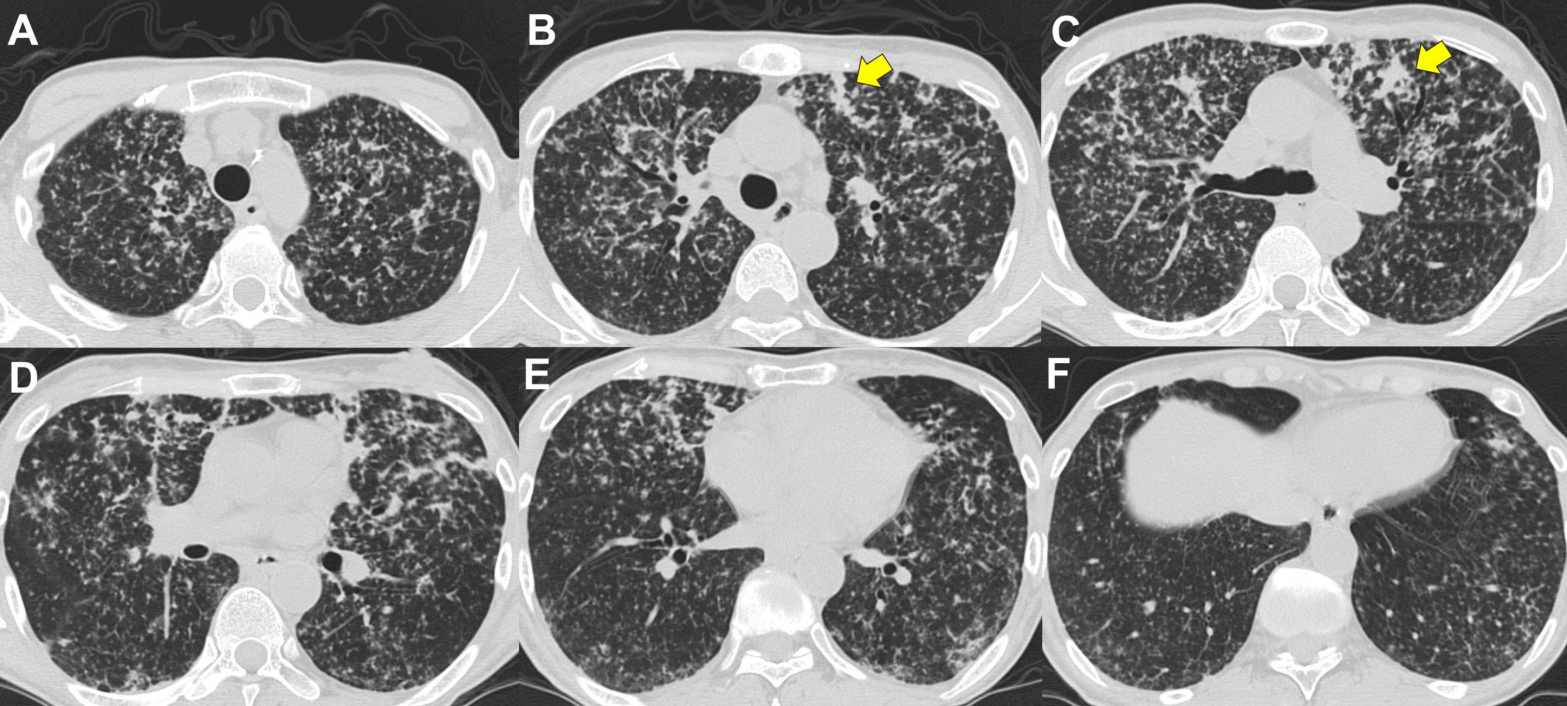
Chest computed tomography Axial thin-section computed tomography images (slice thickness, 0.6 mm; interslice gap, 0.6 mm) demonstrate diffuse centrilobular nodules predominantly involving the upper to middle lung zones of both lungs, corresponding to the reticulonodular opacities observed on the chest radiograph. Some of the nodules show partial confluence (yellow arrows in B and C). Nodules distributed along the bronchovascular bundles are also noted (yellow arrow in B), raising lymphatic involvement as a differential consideration. No focal mass lesions, lobar consolidation, or significant lymphadenopathy are present.

Plain CT of the chest confirmed diffuse reticulonodular patterns distributed throughout both lungs, without focal mass lesions, lobar consolidation, or significant lymphadenopathy (Figure 2).

Based on these imaging findings, hypersensitivity pneumonitis was initially suspected. To establish a diagnosis, transbronchial lung biopsies were performed repeatedly; however, histological examination revealed only non-specific alveolitis, and no definitive diagnosis was obtained. Microbiological studies, including bacterial and fungal cultures, were negative. Laboratory evaluation showed an elevated soluble interleukin-2 receptor (sIL-2R) level of 1,660 U/mL (reference range: 145-519 U/mL), which was the only abnormal tumor marker and raised concern for a possible neoplastic process. All other tumor markers were within normal limits. In addition, angiotensin-converting enzyme and lysozyme levels were normal, and serum fungal markers, including β-D-glucan and cryptococcal antigen, were negative. Given the persistent diagnostic uncertainty, driven primarily by the elevated sIL-2R level, malignant conditions such as lymphoma and carcinomatous lymphangitis remained in the differential diagnosis.

**Figure 3:**
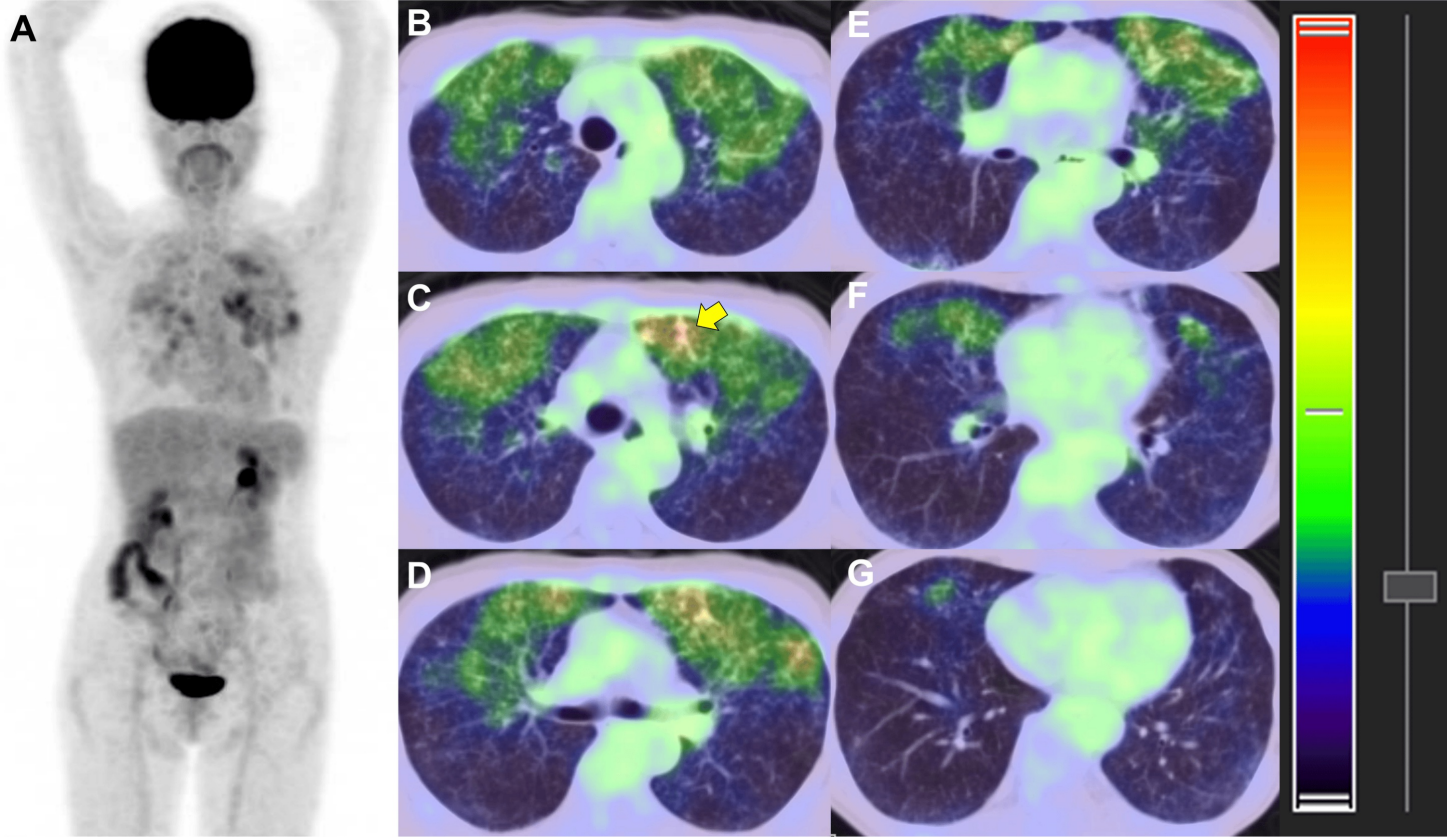
[¹⁸F]FDG-PET/CT (A) Maximum intensity projection (MIP) image demonstrates diffuse increased [¹⁸F]FDG uptake throughout both lungs. (B-G) Fused positron emission tomography/computed tomography (PET/CT) images show increased uptake corresponding to the distribution of the diffuse centrilobular nodules on CT. The most intense uptake is observed in areas of nodular confluence in the left lingula (yellow arrow), with a maximum standardized uptake value of 3.8. For both the MIP and axial images, the standardized uptake value display range was set from 0 to 4, and fused images were displayed with 70% CT and 30% PET weighting. [¹⁸F]FDG: 2-deoxy-2-[¹⁸F]fluoro-D-glucose

Subsequently, [¹⁸F]FDG-PET/CT was performed to further evaluate disease activity and distribution. The examination demonstrated diffuse increased pulmonary uptake confined to the lung parenchyma, with a maximum standardized uptake value of 3.8. No abnormal uptake was observed outside the lungs (Figure 3).

**Figure 4:**
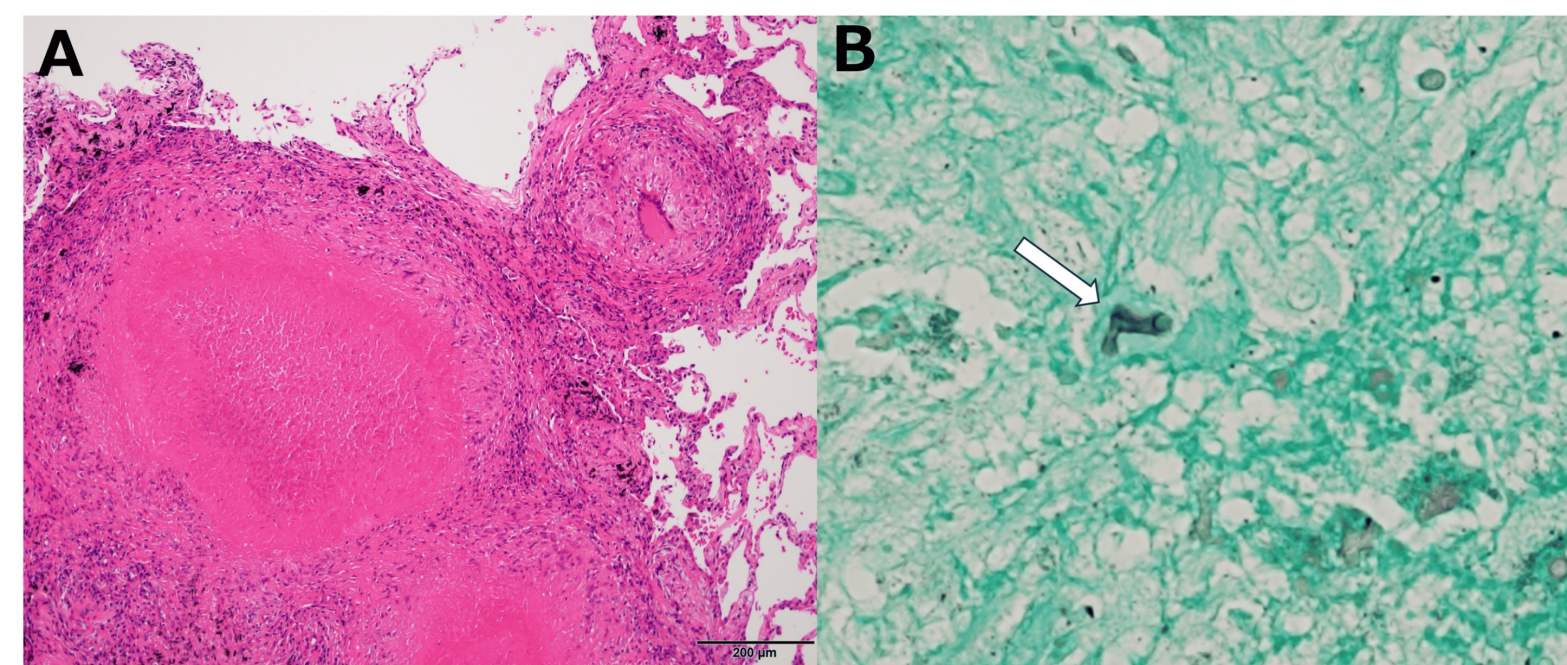
Histopathological findings of the surgically resected lung specimen (A) Hematoxylin and eosin staining shows numerous epithelioid granulomas predominantly located within the lumina of membranous bronchioles. (B) Grocott staining reveals only a few fungal elements (white arrow) within the necrotic centers of the granulomas. The marked discrepancy between the extent of granulomatous inflammation and the minimal fungal burden supports the diagnosis of bronchocentric granulomatosis.

Because a definitive diagnosis could not be established by bronchoscopic biopsy, video-assisted thoracoscopic wedge resection of the right middle lobe was ultimately performed. Histopathological examination of the resected specimen revealed numerous epithelioid granulomas predominantly located within the lumina of membranous bronchioles on hematoxylin and eosin staining. Grocott staining demonstrated only a few fungal elements within the necrotic centers of the granulomas. Species-level identification was not possible based on morphology alone; however, filamentous fungi were suspected. Based on these pathological findings, a diagnosis of BCG induced by a fungal infection was made (Figure 4).

**Figure 5:**
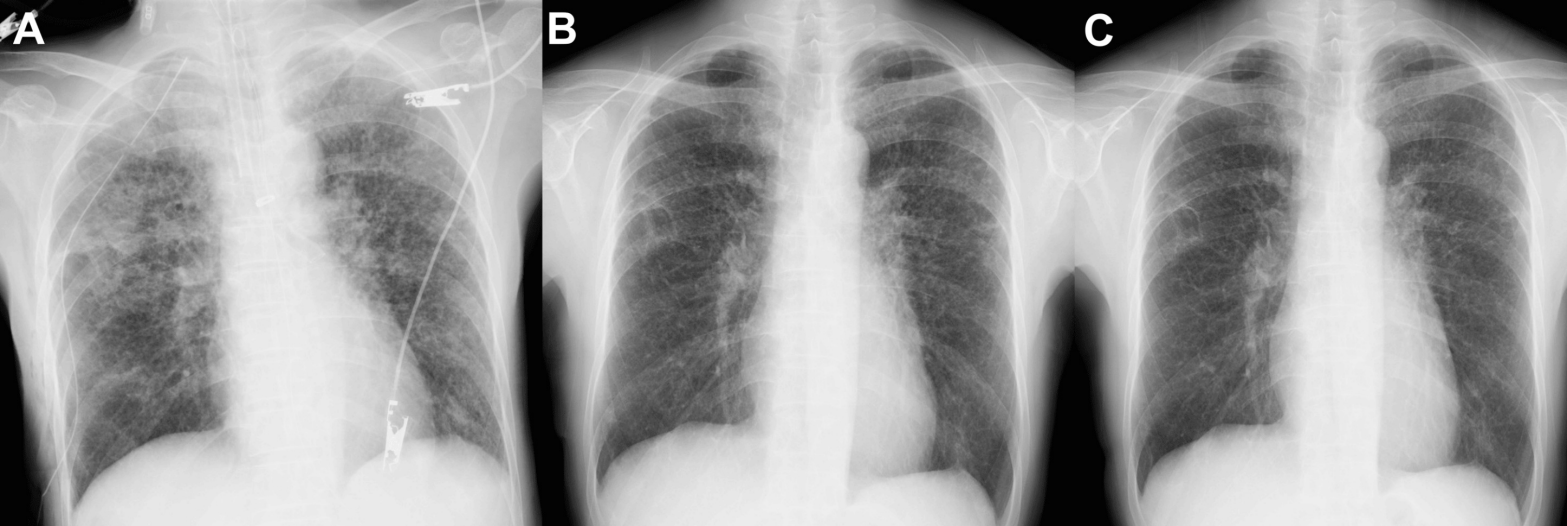
Follow-up chest radiographs Serial chest radiographs obtained immediately after surgery (A), two weeks after surgery (B), and three months after surgery (C) demonstrate gradual improvement of the diffuse reticulonodular opacities in both lungs.

While awaiting the pathological results after biopsy, follow-up chest radiographs already demonstrated improvement in the pulmonary findings, and the patient’s clinical symptoms also gradually resolved. As only a mild cough persisted, she was managed conservatively with antitussive medication alone, and corticosteroid therapy was not initiated. The clinical course was favorable, with no evidence of disease progression. Follow-up chest radiographs obtained immediately after surgery, at two weeks, and at three months demonstrated gradual improvement of the reticulonodular opacities (Figure 5).

## Discussion

BCG is a rare airway-centered granulomatous disorder characterized histopathologically by necrotizing granulomas involving bronchi and bronchioles [1-3]. BCG is considered an extremely rare disease; however, its true epidemiology remains unclear because of the limited number of reported cases [1-3]. Even in recent years, despite advances in imaging diagnostics, reported cases remain scarce. It is presumed that BCG may often resolve spontaneously, as observed in the present case. Consequently, cases in which a definitive diagnosis is established through histopathological confirmation may be exceptionally uncommon. From a radiological perspective, BCG is notable for its wide spectrum of imaging appearances and the absence of disease-specific findings, which frequently leads to diagnostic difficulty.

Based on the very limited number of published reports, the most commonly described CT patterns of BCG include mass-like lesions, solitary or a small number of nodules, and lobar or segmental consolidation with or without atelectasis [4,6]. These imaging features often mimic malignant or infectious lung diseases [5]. In contrast, the present case demonstrated diffuse bilateral reticulonodular opacities, representing an atypical radiological manifestation of BCG. Although a diffuse nodular pattern has been rarely mentioned in previous reports, to our knowledge, no prior studies have clearly demonstrated corresponding CT findings. In this regard, the present case expands the recognized radiological spectrum of BCG by providing detailed CT documentation of this pattern.

The diffuse reticulonodular appearance observed in this case is best explained by the pathological distribution of numerous epithelioid granulomas predominantly located within the lumina of membranous bronchioles. When granulomatous inflammation is diffusely distributed at the level of peripheral airways rather than forming confluent lesions, it may manifest radiologically as multiple small nodules instead of focal masses or lobar consolidation. Given that BCG represents an immunologically mediated response to inhaled antigens rather than active infection, diffuse transbronchial dissemination of antigens may plausibly lead to widespread airway-centered inflammatory reactions. This mechanism provides a reasonable explanation for the diffuse reticulonodular imaging pattern observed in the present case and underscores the importance of radiologic-pathologic correlation.

From an imaging standpoint, the differential diagnosis of diffuse reticulonodular opacities includes hypersensitivity pneumonitis, sarcoidosis, lymphomatoid granulomatosis, and carcinomatous lymphangitis. In the present case, the absence of ground-glass opacities, lymphadenopathy, vascular involvement, and interlobular septal thickening made these diagnoses less likely. This observation emphasizes that BCG should be included in the imaging differential diagnosis of diffuse nodular lung disease, particularly when lesions exhibit an airway-centered distribution.

In infectious and inflammatory lung diseases, imaging findings may vary depending on the presence of underlying conditions. BCG is most commonly associated with underlying conditions such as connective tissue diseases [7,9], asthma [10], and allergic bronchopulmonary aspergillosis [3,11], although recent reports have described its occurrence in other settings, including immunosuppressive therapy, drug-induced reactions [12], and hematologic disorders [13]. These associations support the concept that BCG represents a stereotyped airway-centered immune response to diverse injurious stimuli rather than a single disease entity.

Although cavitation has been rarely reported in BCG [4], it appears to be more common in cases associated with rheumatoid arthritis [7-9], suggesting a possible influence of altered immune status. In such cases, differentiation from chronic pulmonary aspergillosis or other chronic infections is particularly difficult on imaging alone, underscoring the need for histopathological confirmation.

The role of [¹⁸F]FDG-PET in BCG remains poorly defined. To date, only one prior case has reported [¹⁸F]FDG-PET findings in BCG, demonstrating uptake within a mass-like lesion [5]. The present case represents the second reported case of [¹⁸F]FDG-PET in BCG and is unique in demonstrating diffuse pulmonary uptake corresponding to a reticulonodular CT pattern. Although focally intense [¹⁸F]FDG uptake was observed in areas of nodular confluence, the overall diffuse pulmonary uptake likely reflects active granulomatous inflammation rather than infectious burden and provides functional imaging support for the extensive airway-centered inflammatory process suggested by CT.

With regard to management, BCG is generally considered to respond favorably to corticosteroid therapy in many cases [14]. Early and accurate diagnosis may allow prompt initiation of steroid treatment, potentially reducing unnecessary invasive procedures and shortening the overall treatment course. However, in the present case, clinical symptoms and radiographic findings improved without corticosteroid therapy, suggesting that spontaneous resolution may occur in some patients. This observation raises the possibility that BCG may be underdiagnosed, particularly in cases that improve without aggressive treatment. From the perspective of diagnostic imaging, awareness of this entity and its atypical imaging manifestations is therefore important, as timely radiologic recognition may contribute to appropriate clinical management.

## Conclusions

In conclusion, BCG can present with diffuse reticulonodular opacities and diffuse pulmonary [¹⁸F]FDG uptake with focal accentuation in areas of nodular confluence, representing an atypical radiological manifestation of this rare disease. The presence of extensive metabolically active lesions despite only minimal fungal elements reflects the immunologically mediated nature of BCG rather than infectious burden. This case expands the recognized radiological spectrum of BCG and underscores the importance of radiologic-pathologic correlation for accurate diagnosis. Awareness of this presentation may facilitate earlier recognition of BCG in patients with diffuse nodular lung disease and inconclusive microbiological findings.
